# Simulation study on the indirect effect of sulfate on the summer climate over the eastern China monsoon region

**DOI:** 10.1038/s41598-021-87832-5

**Published:** 2021-04-15

**Authors:** Dongdong Wang, Bin Zhu, Hongbo Wang, Li Sun

**Affiliations:** 1 Institute of Atmospheric Environment, China Meteorological Administration, Shenyang, 110166 China; 2 Key Laboratory for Aerosol-Cloud-Precipitation of China Meteorological Administration, Nanjing University of Information Science and Technology, Nanjing, 210044 China; 3 Collaborative Innovation Center on Forecast and Evaluation of Meteorological Disasters, Nanjing University of Information Science and Technology, Nanjing, 210044 China; 4 Key Laboratory of Meteorological Disaster, Ministry of Education (KLME), Nanjing University of Information Science and Technology, Nanjing, 210044 China; 5 Joint International Research Laboratory of Climate and Environment Change (ILCEC), Nanjing University of Information Science and Technology, Nanjing, 210044 China; 6 Liaoning Weather Modification Office, Shenyang, 110166 China

**Keywords:** Attribution, Environmental impact

## Abstract

In this study, we designed a sensitivity test using the half number concentration of sulfate in the nucleation calculation process to study the aerosol-cloud interaction (ACI) of sulfate on clouds, precipitation, and monsoon intensity in the summer over the eastern China monsoon region (ECMR) with the National Center for Atmospheric Research Community Atmosphere Model version 5. Numerical experiments show that the ACI of sulfate led to an approximately 30% and 34% increase in the cloud condensation nuclei and cloud droplet number concentrations, respectively. Cloud droplet effective radius below 850 hPa decreased by approximately 4% in the southern ECMR, while the total liquid water path increased by 11%. The change in the indirect radiative forcing due to sulfate at the top of the atmosphere in the ECMR during summer was − 3.74 W·m^−2^. The decreased radiative forcing caused a surface cooling of 0.32 K and atmospheric cooling of approximately 0.3 K, as well as a 0.17 hPa increase in sea level pressure. These changes decreased the thermal difference between the land and sea and the gradient of the sea-land pressure, leading to a weakening in the East Asian summer monsoon (EASM) and a decrease in the total precipitation rate in the southern ECMR. The cloud lifetime effect has a relatively weaker contribution to summer precipitation, which is dominated by convection. The results show that the ACI of sulfate was one possible reason for the weakening of the EASM in the late 1970s.

## Introduction

Sulfate is one of the most important chemical constituents of aerosols in China and even in East Asia, whose main source is sulfur dioxide (SO_2_, a precursor gas of sulfate) emitted by human activity, such as the burning of coal, oil, and other fossil fuels^1,2^. Sulfate aerosol particles can change the radiation balance by directly reflecting and scattering incident solar radiation; this effect is referred to as the aerosol-radiation interaction (ARI)^3^. In addition, hygroscopic sulfate aerosol can participate in cloud microphysical processes by acting as cloud condensation nuclei (CCN), affecting cloud formation, precipitation, and climate^4^. Increased aerosol can lead to an increase in the concentration of CCN and cloud droplets, resulting in a reduction in the effective radius of the cloud droplets and variations in the cloud optical properties, which is known as the aerosol-cloud interaction (ACI) (also known as the first indirect effect or cloud albedo effect)^5^. On the other hand, if the water vapor remains the same, more hygroscopic aerosol particles will compete for the limited water vapor^6^. This would weaken the collision-coalescence process and inhibit precipitation, prolonging the lifetime of clouds, which will cause the reflection of more solar radiation and reduce the radiative energy at the surface (known as the second indirect effect or cloud lifetime effect)^7^. Previous studies have shown that aerosols with particle geometric diameters greater than 0.05 μm, especially sulfate aerosol particles, contribute the majority of the CCN and ice nuclei to form cloud droplets and ice crystals^8^. The ACI affects the climate by influencing clouds. The radiative forcing of the ACI was assessed to be − 0.70 W·m^−2^ with an uncertainty level that ranged from − 1.8 to − 0.3 W·m^−2^, which may have a greater impact on the climate and a higher uncertainty than the ARI^9^.

The ACI is of great importance in the climate system and play a significant role in climate change^6^. Although measures have been taken to control this factor in recent years, the emission of sulfate and its precursor gas in East Asia is increasingly severe^10,11^. There are a number of studies on radiative forcing and the climatic effects of sulfate aerosols in East Asia^12,13^. However, due to the rather complex relationship between aerosol particles and cloud optical properties, as well as differences in the parameterization schemes of different models, there is considerable uncertainty associated with the study of the ACI^14,15^, especially the cloud lifetime effect^9,16^.

Numerous studies have shown that anthropogenic aerosols may be the possible cause of the weakening trend^17–19^ and the reduction in precipitation^20,21^ of the East Asian summer monsoon (EASM) since the late 1970s. The resulting changes in monsoon intensity and precipitation are derived from the combination of ARI and ACI of anthropogenic aerosols^22^. However, most previous studies have focused on ARI while studies on the ACI on the East Asian monsoon and precipitation are still rare. Even if the studies involving the ACI are typically based on the overall effects of aerosols, there have been few studies that distinguish the ACI itself. However, this type of analysis is useful to determine the contribution of various effects and understand the mechanisms of aerosol effects^4,15^. In previous studies, the method commonly used to study the ACI was the “parameterization” or “emission source” methods. The "parameterization" method refers to the introduction of a parameter formula for the relationship between cloud droplets and aerosols in the model. Compared to results without parameters, the “parameterization” model can obtain the ACI^6,13,23^. The “emissions source” method is mostly used in the most recent models containing the mechanisms between aerosols and cloud droplets, comparing the results of simulations using emission sources from different periods (usually representing present day and pre-industrial) to obtain the ACI^17,22,24^. The latter, however, often results in difficulties in distinguishing between the ARI and ACI.

To distinguish the ARI and ACI that aerosols have on the East Asian monsoon, we adopted a special "parameterization" method. By setting the sulfate aerosol optical depth (AOD) to zero during the calculation process to turn off its ARI, while the sulfate aerosol concentration does not change (theoretically) without changing the ACI, we analyzed the ARI of sulfate on the subseasonal march of the East Asian subtropical summer monsoon^25^. Next, we continued to use this method by modifying an aerosol nucleation effect, attempting to study the impact that the ACI has on the EASM without changing the ARI. In this study, we aim to investigate the ACI of sulfate on summertime clouds, precipitation, and monsoon intensity over the eastern China monsoon region (ECMR) with the Community Atmosphere Model version 5 (CAM5). We expect the results to provide a reference for the quantitative study of the mechanism(s) and possible contribution of the ACI. In this study, the ACI of sulfate and its impact on the regional climate of China are investigated in the Results section. A summary is presented in the Conclusions and discussion section. Finally, the model and data are briefly introduced, and the experiment designs are described in the Methods section.

## Results

### Climatological distribution of the EASM

**Figure 1:**
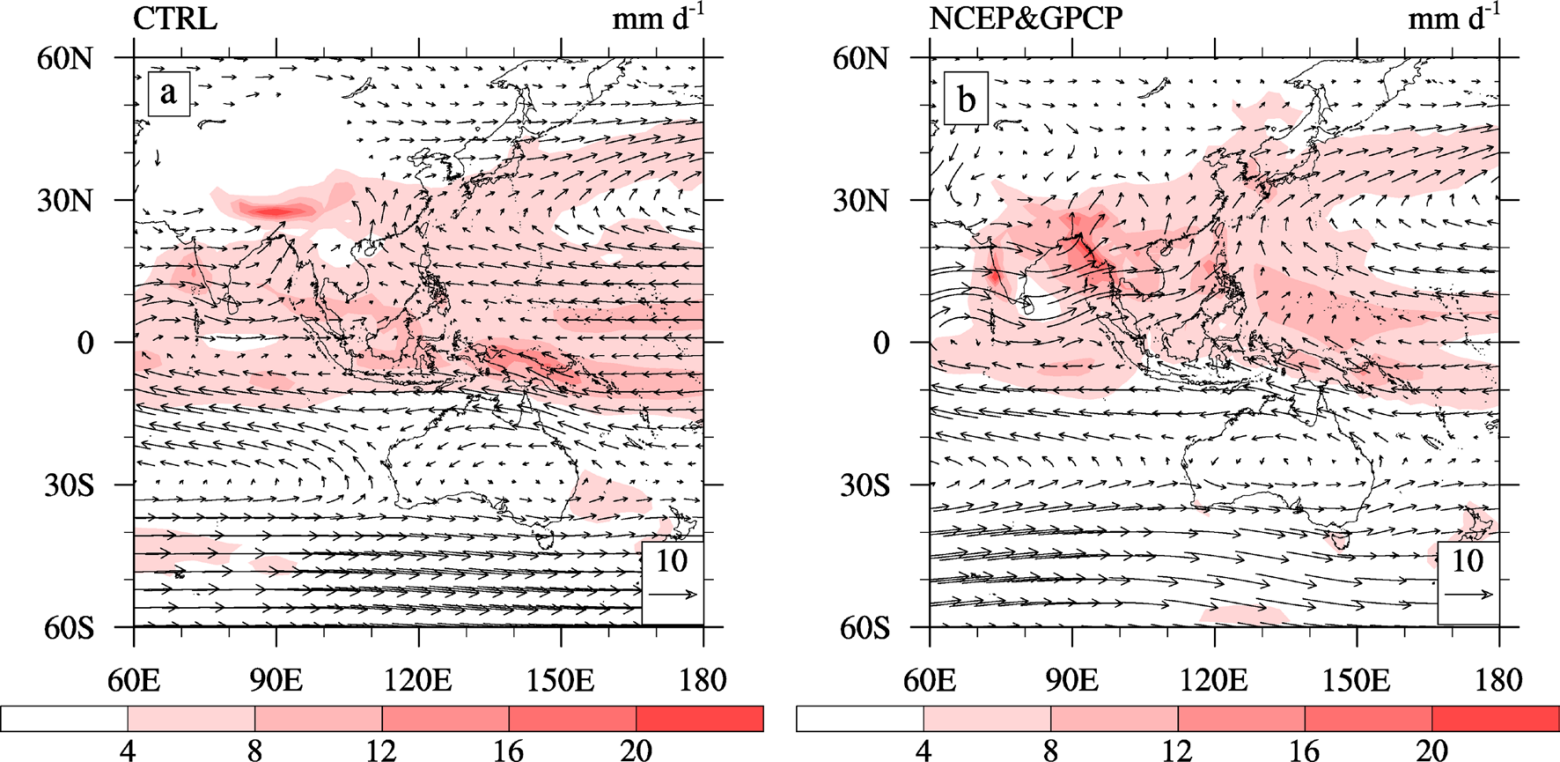
Horizontal distributions of the total precipitation rate (shading; units: mm·d^−1^) and 850 hPa wind (vectors; units: m·s^−1^) during the summer: (**a**) CTRL and (**b**) NCEP and GPCP. The software used to draw the map was NCL Version 6.2.1^35^.

CAM5 has been extensively evaluated on modeled aerosol and clouds^25–27^ and used to study the effects that aerosols have on climate^28–30^. To examine the capability of CAM5 to simulate atmospheric circulation and precipitation over East Asia during the summer (June, July, and August, or JJA). Figure 1 shows comparisons of the mean simulated JJA 850 hPa wind vectors and precipitation rate over the Asian monsoon regions with observations. The 850 hPa wind observation data derives from the National Centers for Environmental Prediction (NCEP) reanalysis^31^ while the precipitation data originates from the Global Precipitation Climatology Project (GPCP) dataset^32^. Our results show that CAM5 reproduces the main summer features of the 850 hPa wind and rain belt spatial distributions, in which we can observe certain biases (Fig. 1). The simulated westerly flows from the Arabian Sea to the South China Sea, as well as southerly flows from the Bay of Bengal to the South China Sea, are weaker than the observations. This results in less precipitation in eastern and southern China due to reduced moisture transport from the oceans. This is a drawback in most atmospheric GCMs when simulating the East Asian summer climate^33,34^. Despite these deficiencies, the model reproduces the spatial pattern of the 850 hPa wind and precipitation over the East Asian monsoon regions well. Thus, CAM5 can appropriately investigate the impact of anthropogenic aerosols on the EASM.

### Summertime sulfate, cloud characteristics and radiative forcing

**Figure 2:**
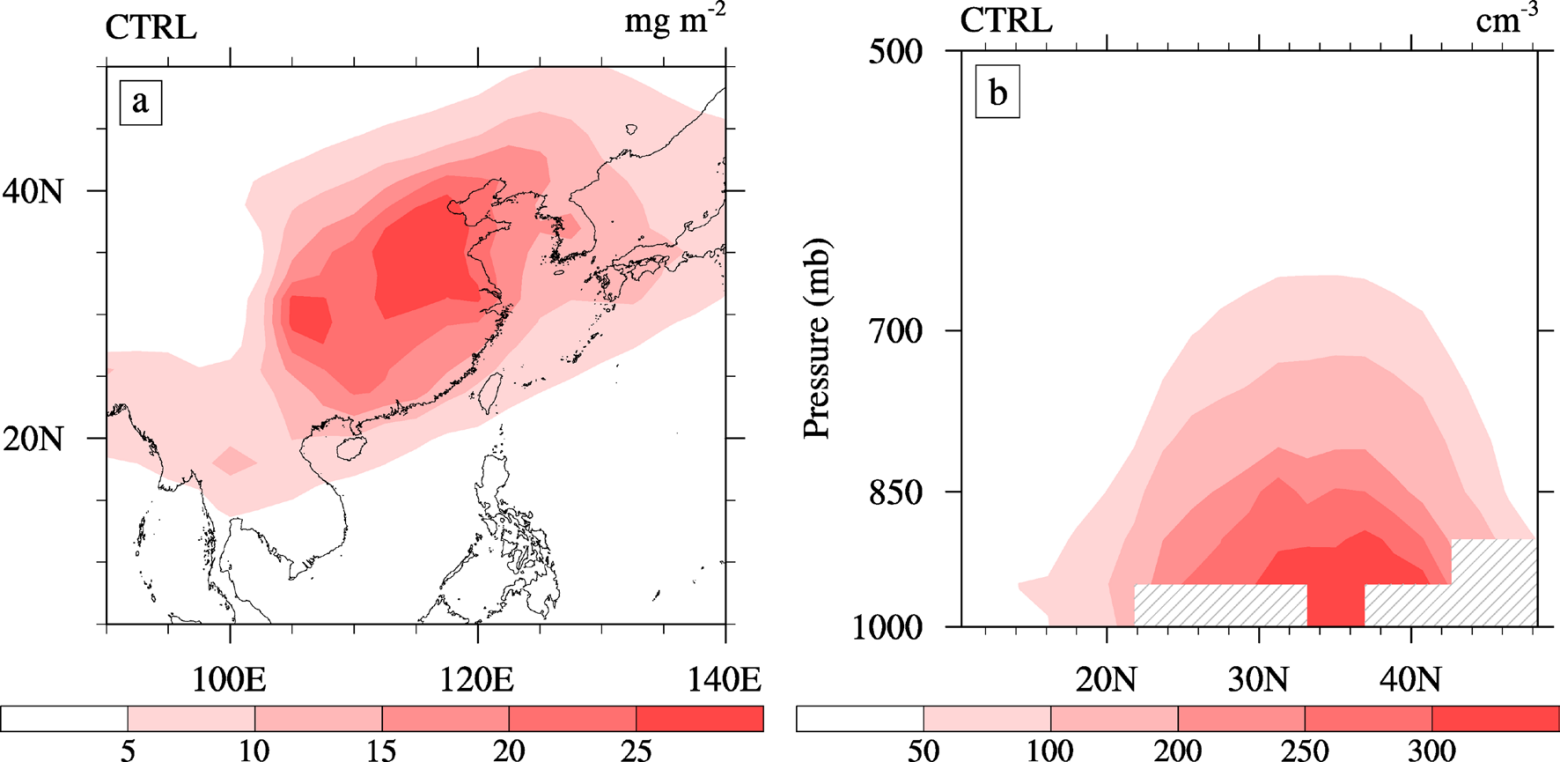
(**a**) Spatial distribution of the sulfate aerosol burden (units: mg·m^−2^) and (**b**) latitude-altitude sections of cloud condensation nuclei concentration at a supersaturation of 0.1% (units: cm^−3^) averaged over 105°–120° E from CTRL during summer. The software used to draw the map was NCL Version 6.2.1^35^.

This study focuses on the effects of aerosols on the climate over the ECMR (defined in this study as 20°–45° N, 105°–120° E). We further divided the monsoon region into the South ECMR (defined as 20°–27.5° N, 105°–120° E), Center ECMR (27.5°–32.5° N, 105°–120° E), and North ECMR (defined as 32.5°–45° N, 105°–120° E) to study the climatic response in different regions to aerosols. Figure 2a shows the distribution of the summer sulfate aerosol burden based on the control experiment (CTRL). We can observe that sulfate is mainly distributed over the ECMR continent and its adjacent oceans, where higher values concentrate in the North China and Sichuan Basin, which are located in the Center ECMR. The maximum values of sulfate aerosol burden exceeded 28 mg·m^−2^. The magnitude and spatial structure of the simulated summertime sulfate aerosol burden are consistent with previous model studies^24,33^. The EASM is characterized by a low-level southern airflow, prevailing over East Asia^36,37^, which facilitates the northward transport of sulfate aerosols and their precursors. As secondary pollutants, sulfates experience more active conversion from SO_2_ gas to particles in summer due to higher solar radiation and stronger photochemical reactions^24^. In spite of the strong wet scavenging process, the greater amount of water vapor, which enhances the hygroscopic growth of sulfate aerosols, also likely results in a higher burden during the summer^6,25^.

Figure 2b shows the latitude-altitude sections of the CCN concentrations at a supersaturation of 0.1% averaged over 105°–120° E from CTRL during summer. The vertical distribution of CCN is generally consistent with the distribution of the sulfate burden. The high CCN value corresponds to the high burden of the Center and North ECMR (Fig. 2a). The CCN are mainly distributed below 700 hPa over the ECMR (20°–45° N). The maximum values occur below approximately 900 hPa to the north of 30°N, with a concentration exceeding 300 cm^−3^. This result is consistent with that reported by Jiang^24^. Table 1 lists the statistics of the changes in the summertime cloud characteristics caused by the ACI of sulfate over the ECMR. Changes in the CCN at 850 hPa due to the ACI of sulfate were 56.19 cm^−3^ (~ 30%) in the ECMR, with the largest changes (~ 32%) in the Center ECMR, where the CCN has the highest concentration.

**Table 1:** Statistics associated with the changes in the summertime CCN concentration at a supersaturation of 0.1% (CCN, in cm^−3^) at 850 hPa, vertically integrated cloud droplet number concentration (VCDNC, in 10^6^·cm^−2^), low-level cloud cover (CLDLOW, in %), high-level cloud cover (CLDHGH, in %), cloud-liquid water path (LWP, in g·cm^−2^), shortwave cloud forcing (SWCF, in W·m^−2^), longwave cloud forcing (LWCF, in W·m^−2^), and shortwave radiative forcing at the TOA of the all-sky (the sky is covered by clouds) (FSTOA, in W·m^−2^) caused by the ACI of sulfate averaged over various regions.

	CCN	VCDNC	CLDLOW	CLDHGH	LWP	SWCF	LWCF	FSTOA
ECMR	56.19	2.69	0.74	− 0.92	13.20	− 3.18	0.53	− 3.74
South ECMR	48.69	2.87	0.23	− 0.73	14.26	− 2.16	0.87	− 4.23
Center ECMR	80.32	4.62	1.84	− 1.12	25.36	− 5.55	0.95	− 6.54
North ECMR	53.35	2.04	0.72	− 0.96	9.12	− 3.77	0.22	− 2.66

Eastern China monsoon region (ECMR, 20°–45° N, 105°–120° E), South ECMR (20°–27.5° N, 105°–120° E), Center ECMR (27.5°–32.5° N, 105°–120° E), and North ECMR (32.5°–45° N, 105°–120° E).

**Figure 3:**
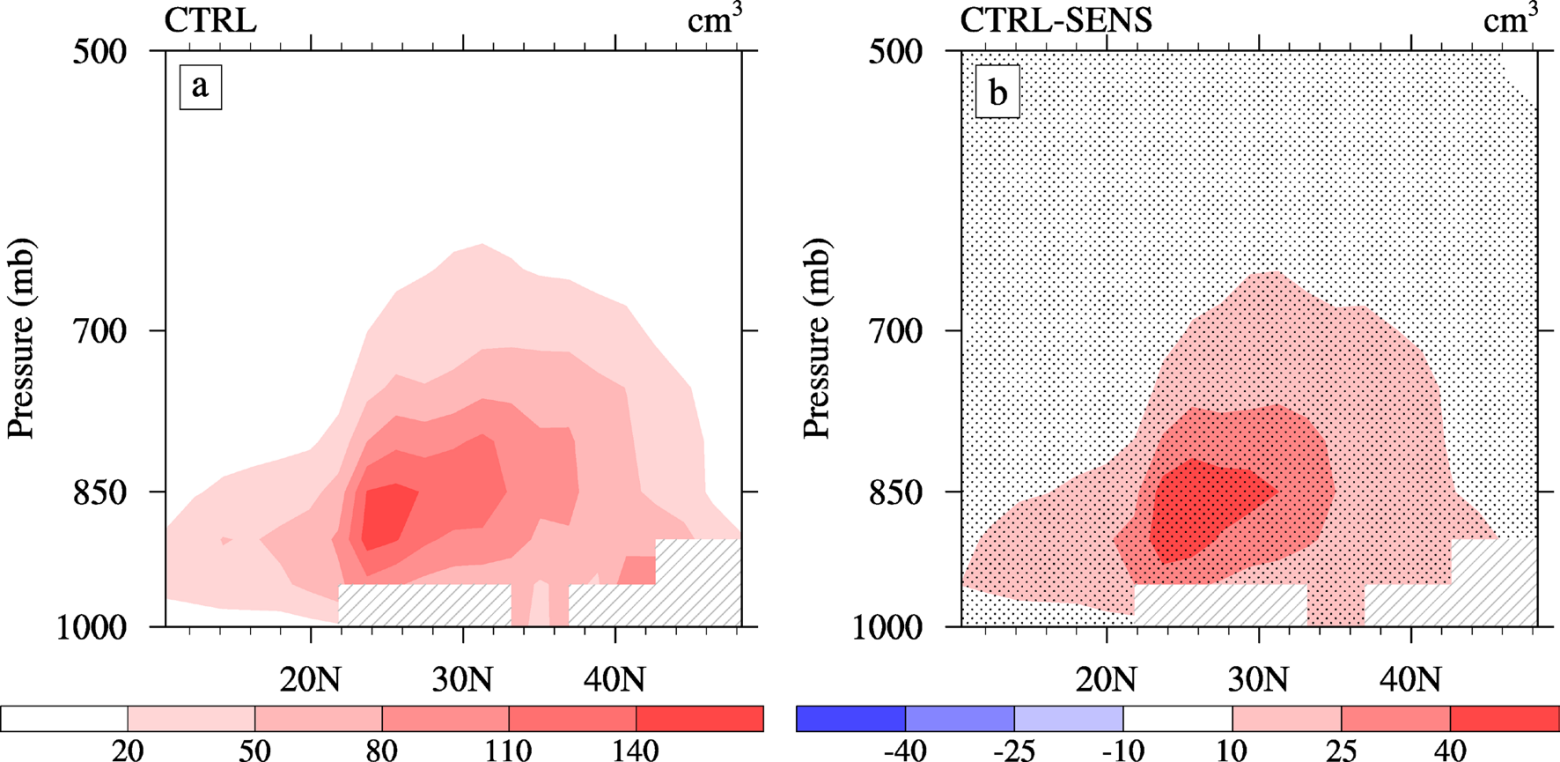
Latitude-altitude sections of the (**a**) in-cloud cloud droplet number concentration (units: cm^−3^) from CTRL and (**b**) changes caused by the ACI of sulfate averaged over 105°–120° E during summer. The dotted areas denote regions of statistically significant changes at a 90% confidence level.

Changes in the number concentration of CCN will result in variations in the cloud droplet number concentration (CDNC)^5^. The vertically integrated CDNC caused by the ACI of sulfate is 2.69 × 10^6^ cm^−2^. Our results are more similar to those reported in Han^38^, who showed that the CDNC caused by ammonium-sulfate-nitrate ranged from 2.4 × 10^6^ to 3 × 10^6^ cm^−2^ in China. Figure 3a, b present the latitude-altitude sections of the in-cloud CDNC during summer from CTRL, as well as their changes due to the ACI of sulfate. The simulated in-cloud CDNC is mainly concentrated over the ECMR (20°–45° N) during summer. The maximum value is below approximately 850 hPa in the South ECMR (approximately 140 cm^−3^). The increase in the CCN due to the ACI of sulfate causes an increase in the vertically integrated and in-cloud CDNC (Table 1 and Fig. 3b). This results in an increase in the vertically integrated CDNC by approximately 34% averaged over the ECMR. The spatial distribution of the CDNC is represented by an elevated increase in the South and Center ECMR.

**Figure 4:**
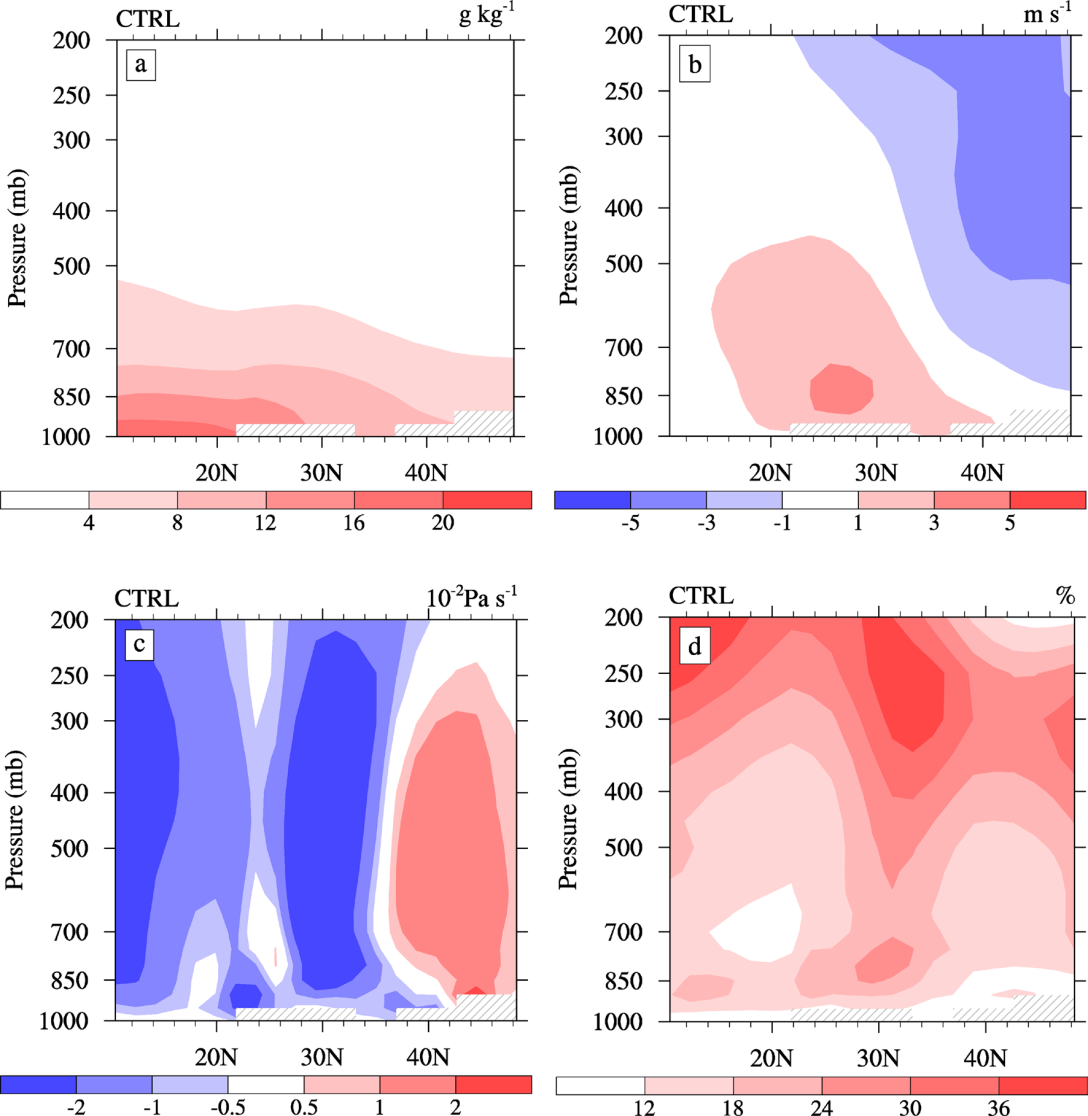
Latitude-altitude sections of the (**a**) water vapor content (units: g·kg^−1^), (**b**) meridional wind (units: m·s^−1^), (**c**) vertical velocities (units: 10^−2^ Pa·s^−1^), and (**d**) cloud cover (in %) averaged over 105°–120° E from CTRL during summer.

However, the vertical distribution of the high-value center of the increased in-cloud CDNC is inconsistent with the CCN, mainly in the Center and North ECMR (Fig. 2b). As a result, we must perform further analysis of the climatic conditions, such as water vapor and wind fields. Figure 4 illustrates the simulated summertime water–vapor content, meridional wind, vertical velocity, and cloud amount from CTRL. As shown in Fig. 4a, in summer, the total water vapor is mainly distributed over the ocean, which is to the south of approximately 20°N and gradually diminishes from the ocean to the land due to low-level southward airflow of the EASM. The summer circulation in East Asia is characterized by a southerly flow in the lower troposphere and a northerly flow in the upper troposphere (Fig. 4b). The maximum southerly wind appears to the south of 30ºN below approximately 700 hPa. In the vertical direction (Fig. 4c), the ocean from the equator to approximately 20°N and the Center ECMR are strong ascending regions, and a strong sinking area appears in the North ECMR. We note that there is also an ascending area over the South ECMR. Such water vapor and vertical circulation characteristics, combined with the distribution of the CCN, result in large amount cloud cover over the Center and South ECMR (Fig. 4d). In summary, the Center and South ECMR have a higher water vapor content and cloud cover in summer^24,38^, which explains why the in-cloud CDNC is higher here, despite a higher CCN in the North ECMR.

**Figure 5:**
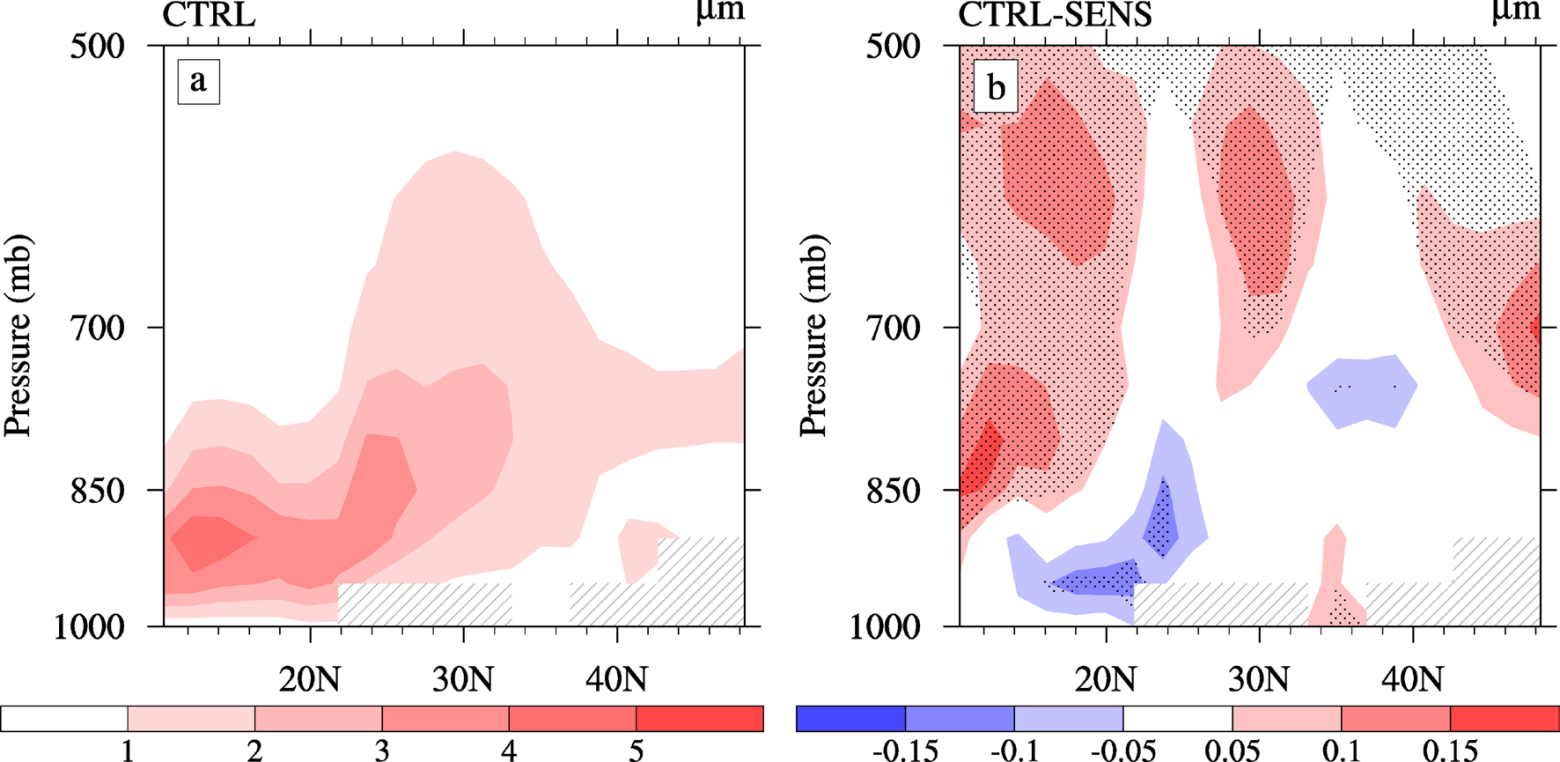
Latitude-altitude sections of the (**a**) cloud droplet effective radius (units: µm) from CTRL and (**b**) changes caused by the ACI of sulfate averaged over 105°–120° E during summer. The dotted areas denote regions of statistically significant changes at a 90% confidence level.

According to the cloud albedo effect, the changes in the CDNC may have an effect on the cloud droplet effective radius (Reff) if the water vapor remains the same, leading to variation in the cloud optical thickness and cloud albedo, which, in turn, affects the radiation balance^5^. Based on Fig. 5a, the highest value of the summertime Reff simulated by the CTRL appears over the ocean (south of 20°N) below 850 hPa (approximately 4.5 µm), and the second highest value (approximately 3.5 µm) occurs in the South and Center ECMR below 700 hPa, related to an abundant water vapor content (Fig. 4a). By comparing Figs. 3a and 5a, the distribution of the Reff over the land of the ECMR agrees with the in-cloud CDNC.

**Figure 6:**
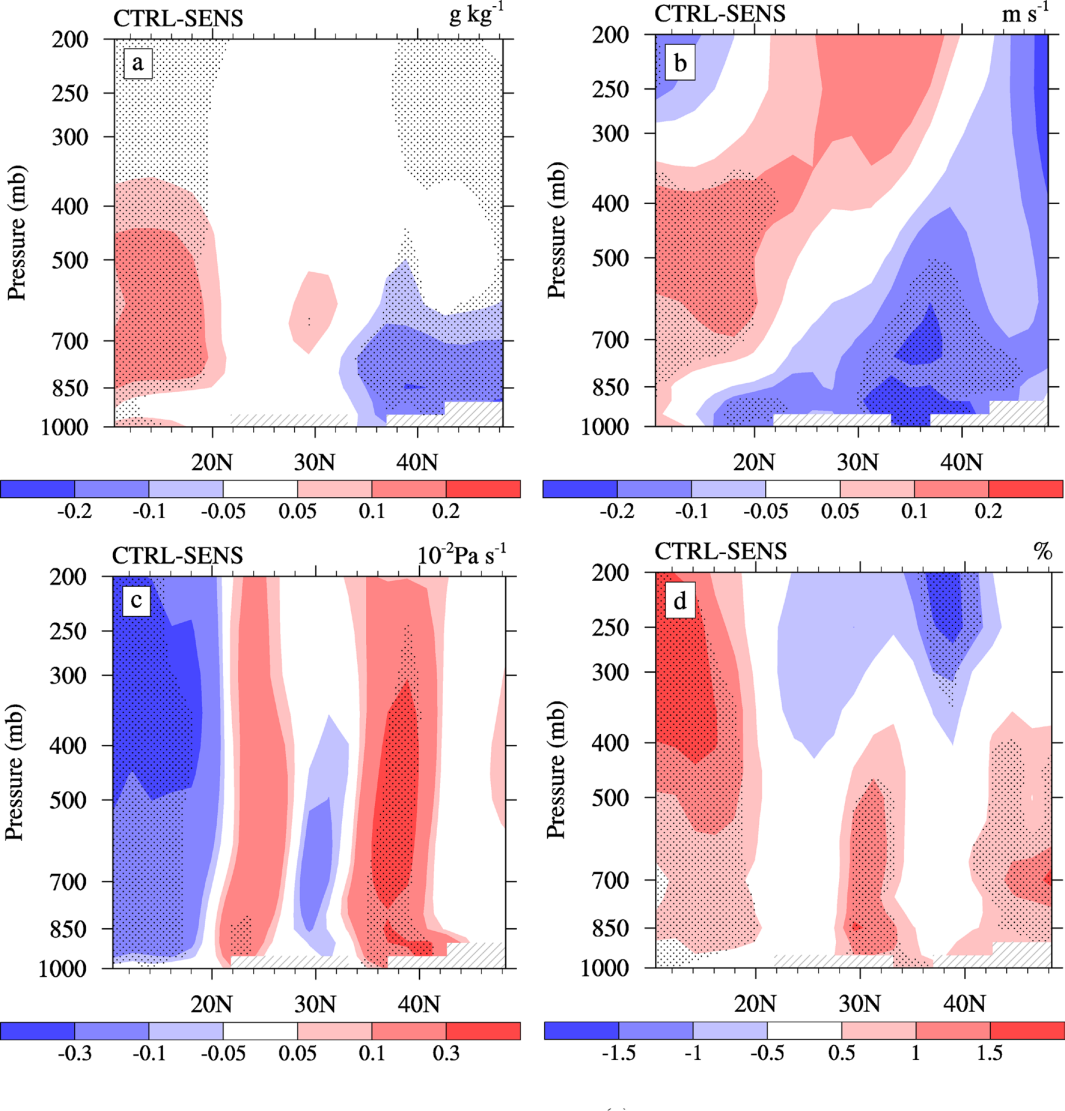
Latitude-altitude sections of changes in the (**a**) water vapor content (units: g·kg^−1^), (**b**) meridional wind (units: m·s^−1^), (**c**) vertical velocities (units: 10^−2^ Pa·s^−1^), and (**d**) cloud cover (in %) averaged over 105°–120° E caused by the ACI of sulfate in summer. The dotted areas denote regions of statistically significant changes at a 90% confidence level.

The changes of Reff are mainly concentrated in the southern coastal area of the ECMR (Fig. 5b). In the South ECMR, the average change in Reff is approximately 0.05 µm, with a maximum change of approximately 0.15 µm (~ 4%) at 850 hPa. An increase in the in-cloud CDNC due to the ACI of sulfate (Fig. 3b) causes the decrease in the Reff below 800 hPa over the South ECMR (Fig. 5b). As shown in Fig. 5b, the Reff also changes over the tropical ocean, as well as both the Center and North ECMR, due to changes in certain conditions, such as the water vapor content and vertical circulation. Figure 6 illustrates the changes in summertime water–vapor content, meridional wind, vertical velocity, and cloud amount caused by the ACI of sulfate. The Reff decreases over the tropical ocean below 850 hPa, but increases above 850 hPa. The decrease in the Reff in the lower layer and the increase above 850 hPa is associated with significant rising motion anomalies (Fig. 6c) and an increased cloud cover (Fig. 6d) caused by changes in the total water vapor content (Fig. 6a). The increase in Reff over the Center ECMR above approximately 800 hPa is due to the same reason. Contrary to changes over the tropical ocean, the Reff over the North ECMR increases below 850 hPa and decreases above 850 hPa due to the descending motion anomalies (Fig. 6c). In addition, Reff increases above 850 hPa to the north of 45ºN, which is related to an increase in cloud cover (Fig. 6d). In general, changes in Reff are not only related to aerosols, but also to the circulation feedback caused by the ACI of aerosols, such as on water vapor and vertical circulation. The changes in Reff are relatively small compared with other studies^6,22^, which may be due to the 2-year low-pass filtering was used to remove the interannual change. In addition, the reduced water vapor content (Fig. 6a) over the North ECMR may be related to the weakening of the southerly wind in the lower layer (Fig. 6b).

**Figure 7:**
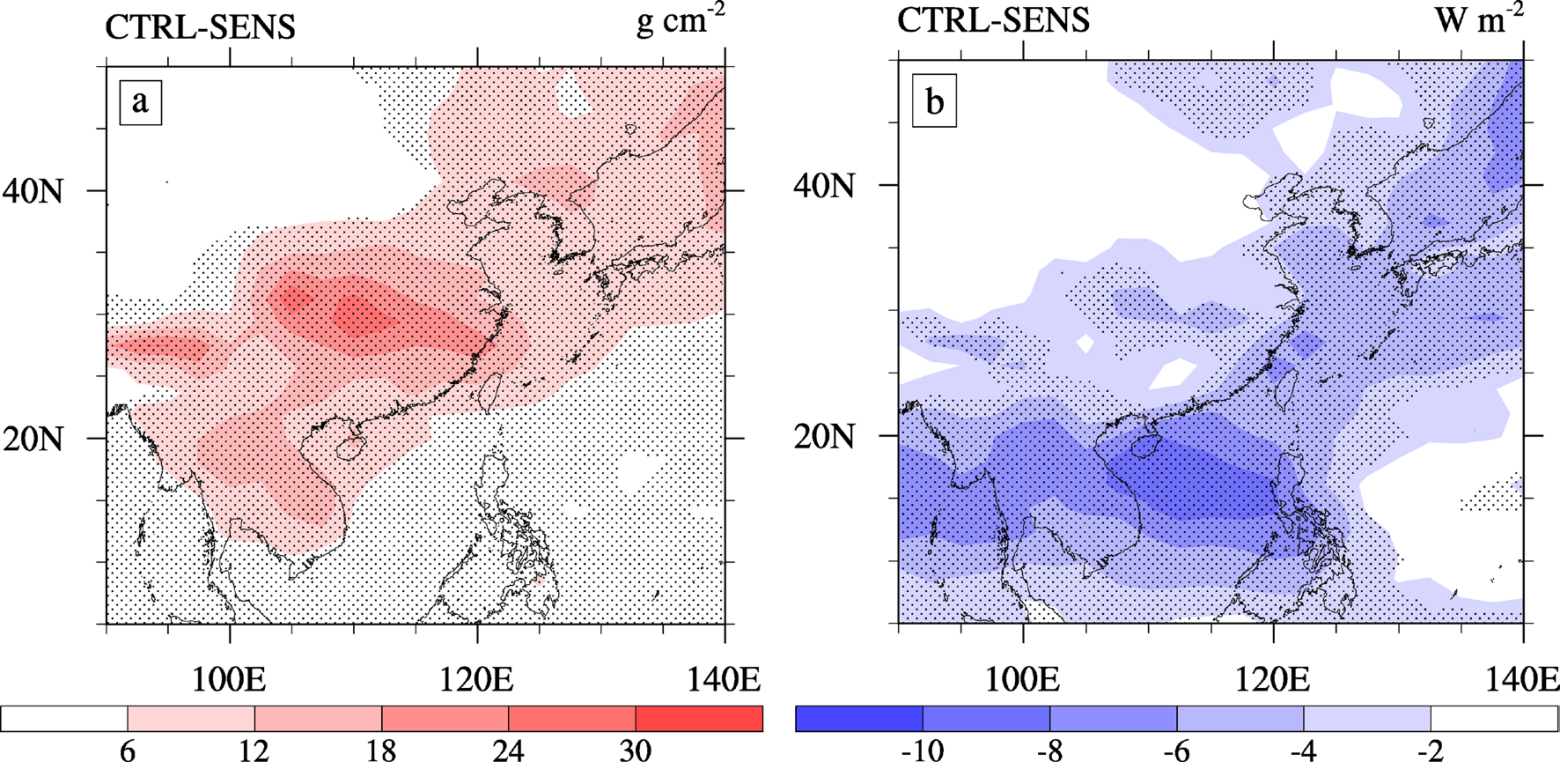
The horizontal distribution of changes in the (**a**) cloud-liquid water path (LWP, units: g·m^−2^) and (**b**) shortwave cloud forcing (SWCF, unit: W·m^−2^) caused by the ACI of sulfate during summer. The dotted areas denote regions of statistically significant changes at a 90% confidence level. The software used to draw the map was NCL Version 6.2.1^35^.

The cloud-liquid water path (LWP) is an important variable for cloud properties that can be affected by the aerosol second indirect effect^24,39^. The increase in the LWP is because an increased amount of smaller droplets may lead to a reduction in the precipitation efficiency, which is related to the aerosol cloud lifetime effect^7^. The distribution of the simulated summer LWP is demonstrated in Fig. 7a. The Center and South ECMR have a higher LWP in the summer while the North ECMR is slightly lower (Table 1). This distribution is mainly related to the distribution of summer rain belts and clouds in East Asia. Due to the ACI of sulfate, the increased LWP in the ECMR by ~ 11%, ~ 10% in the South and North ECMR, and ~ 15% in the Center ECMR. The areas with higher LWP showed more significant increases.

Smaller cloud droplets and larger LWP lead to an increase in cloud albedo. The shortwave cloud forcing (SWCF) and longwave cloud forcing (LWCF) were calculated based on the difference at the top of the atmosphere (TOA) between all-sky (the sky is covered by clouds) and clear-sky conditions, which is a cloud property related to the radiation balance process within the climate system^24^. The spatial distribution and seasonal characteristics of SWCF are typically related to the LWP, as illustrated in Fig. 7b. The increase in cloud albedo and cloud cover results in an enhancement of SWCF (more negative). As listed in Table 1, the ACI of sulfate strengthens the SWCF over the ECMR by − 3.18 W·m^−2^, with the largest change also in the Center ECMR by − 5.55 W·m^−2^, which is consistent with the variation characteristics of the LWP. The magnitude and spatial structure of the changes in the SWCF are consistent with that reported in Jiang^22^ by − 5.5 W·m^−2^. The summertime all-sky shortwave radiation forcing at the TOA is − 3.74 W·m^−2^ over the ECMR. Chen^6^ showed that the summertime first indirect radiation forcing is − 3.04 W·m^−2^. Our estimates are slightly higher because we also considered the cloud lifetime effect. The increased LWCF (positive) is related to reduced high clouds, which is smaller than the SWCF.

### The changes of summertime temperatures and sea level pressure

Incoming solar radiation may be absorbed at the surface and within the atmosphere by aerosols, water vapor, clouds, and other trace gases. In the simulation design of this study, we assumed that there was no change in the gas factor. However, changes in the radiation balance are bound to cause changes in factors, such as circulation, water vapor, and clouds. Therefore, when considering temperature changes, we must consider all-day conditions, i.e., changes in the presence of water vapor and clouds. In general, changes in the surface air temperature are due to the all-sky net shortwave flux, net longwave flux, sensible heat flux, and latent heat flux at the surface^22^. Table 2 lists the changes in the heat fluxes and air temperature at the surface due to the ACI of sulfate during summer. We observe that the ACI of sulfate leads to a decrease in the net shortwave flux at the surface averaged over the ECMR, with the largest change in the Center, which is consistent with the variation in the SWCF. The ACI of sulfate results in a decrease in the net longwave and sensible heat fluxes at the surface, whose value is small. The surface latent heat flux is reduced to approximately half of the net shortwave flux and remains the largest in the Center ECMR.

**Table 2:** Changes in heat fluxes at the surface, surface air temperature, and sea level pressure caused by the ACI of sulfate averaged over the ECMR during summer.

	Net shortwave flux (W·m^−2^)	Net longwave flux (W·m^−2^)	Sensible heat flux (W·m^−2^)	Latent heat flux (W·m^−2^)	Surface air temperature (K)	Sea level pressure (hPa)
ECMR	− 3.81	− 0.75	− 0.76	− 1.68	− 0.32	0.17
South ECMR	− 4.20	− 0.49	− 0.83	− 1.56	− 0.14	0.05
Center ECMR	− 6.97	− 1.71	− 1.76	− 3.46	− 0.32	0.18
North ECMR	− 2.69	− 0.63	− 0.43	− 1.24	− 0.42	0.23

**Figure 8:**
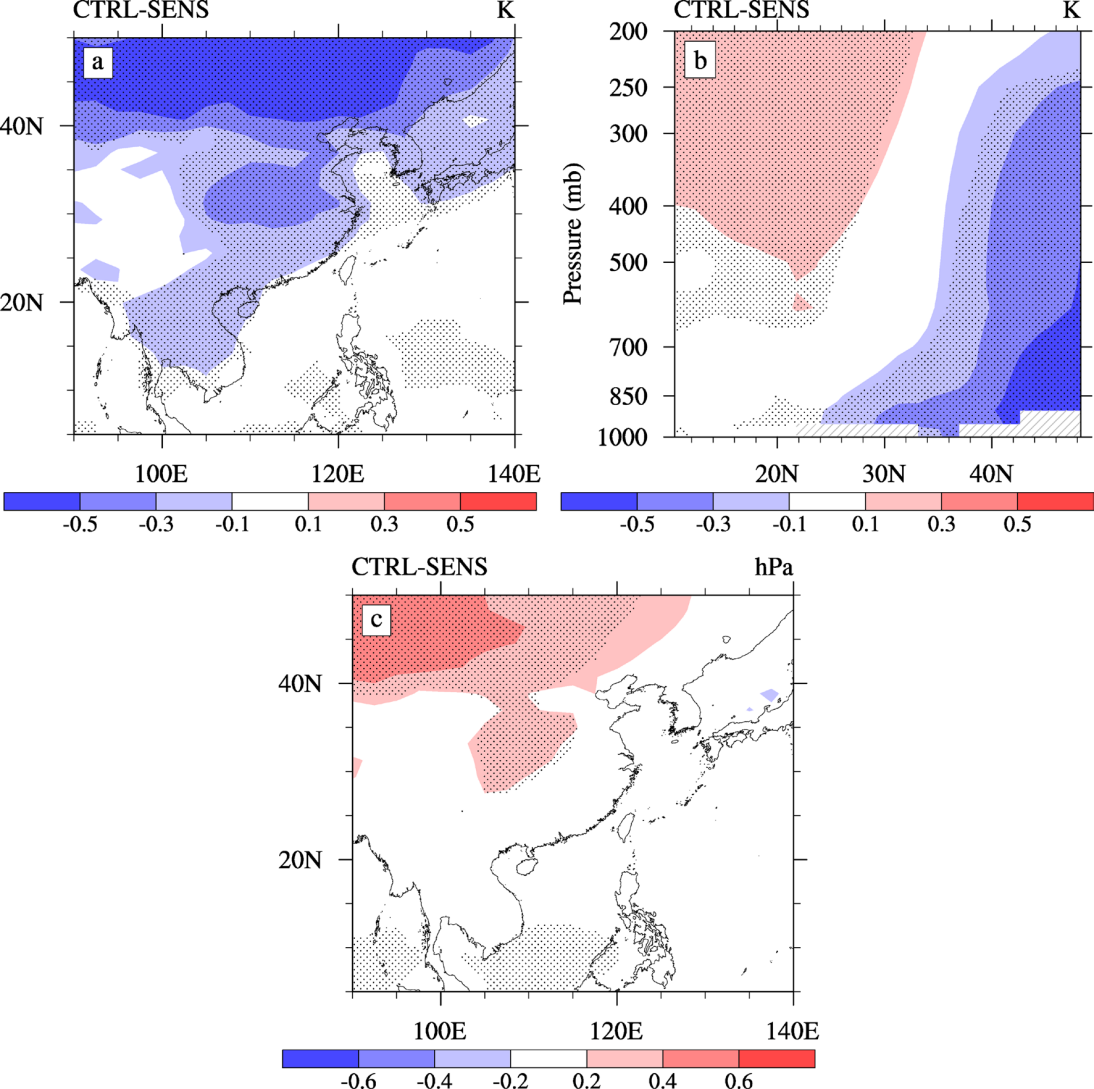
The horizontal distribution of changes in the (**a**) surface temperature (units: K), (**c**) sea level pressure (units: hPa), and latitude-altitude sections of the changes in the (**b**) atmospheric temperature (unit: K) caused by the ACI of sulfate averaged over 105°–120° E during summer. The dotted areas denote regions of statistically significant changes at a 90% confidence level. The software used to draw the map was NCL Version 6.2.1^35^.

**Figure 9:**
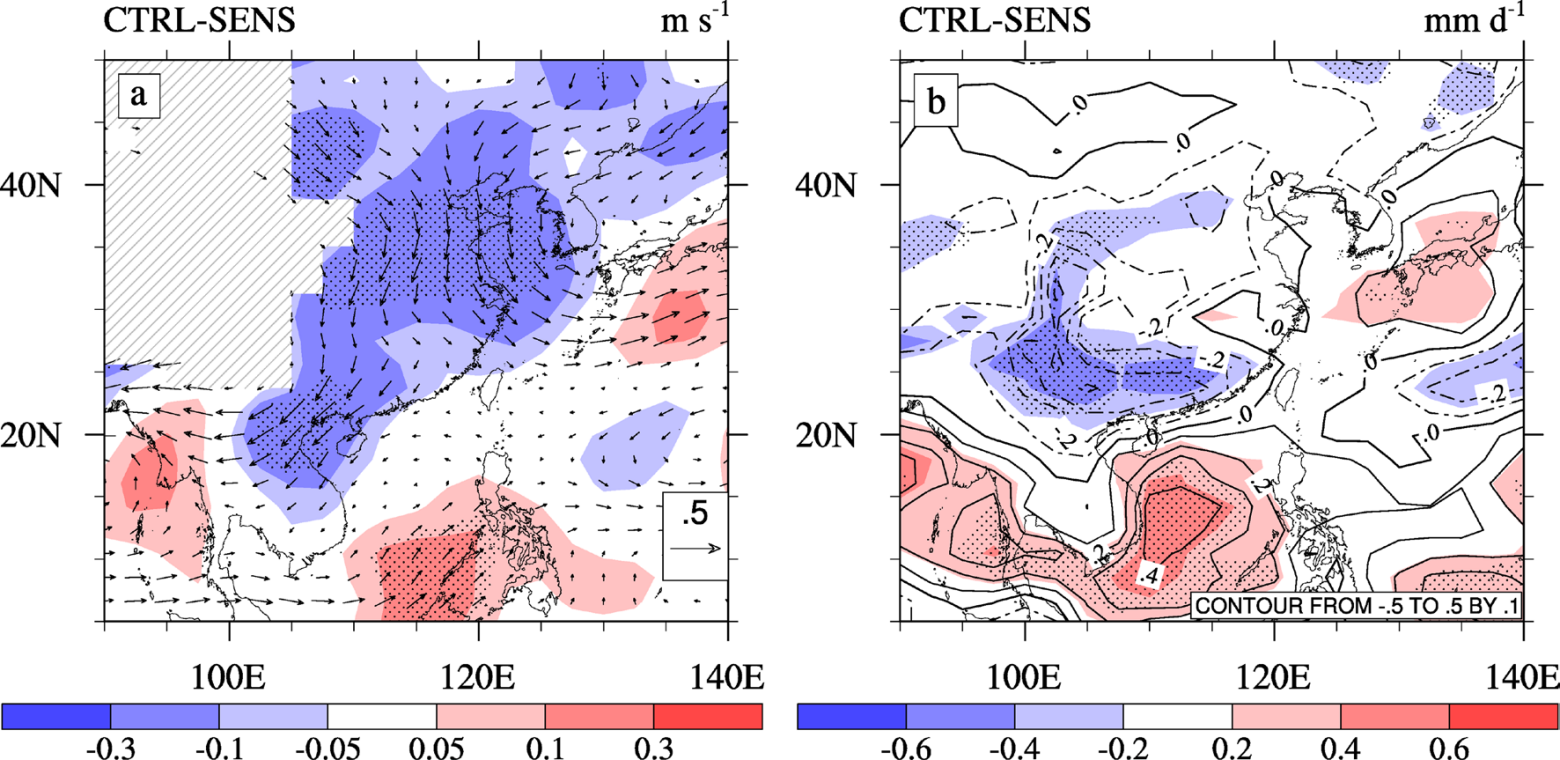
The horizontal distribution of changes in the (**a**) 850 hPa wind (vectors; units: m·s^−1^) and meridional wind (shading; units: m·s^−1^), (**b**) total precipitation rate (shading; units: mm·d^−1^) and convective precipitation rate (contours; units: mm·d^−1^) caused by the ACI of sulfate in summer. The dotted areas denote regions of statistically significant changes at a 90% confidence level. The software used to draw the map was NCL Version 6.2.1^35^.

Combined with the analysis of the heat fluxes at the surface, Fig. 8 displays the changes in the summertime surface air temperature and sea level pressure caused by the ACI of sulfate. As shown in Fig. 8a, the ACI of sulfate leads to a significantly large surface cooling over the ECMR while increased cooling occurs in the North and Center ECMR. This result is consistent with that reported in Jiang^22,24^. The change in the surface air temperature mainly depends on variations in the net shortwave and latent heat fluxes. It is worth noting that there is a deep cooling north of 40°N, which cannot be completely explained by the change in heat flux. This cooling is caused by temperature advection due to changes in circulation (increased northerly wind, as shown in Fig. 9a). Figure 8b shows the change in vertical temperature caused by the ACI of sulfate. Corresponding to the change in the surface air temperature, the cooling emerges north of 24°N, where it is shallower to the south of approximately 34°N and deeper in the north. Atmosphere cooling is ~ 0.3 K.

In the Northern Hemisphere summer, the land area over East Asia is generally a low-pressure area while the eastern adjacent ocean is a high-pressure area due to the western Pacific subtropical high. With the changes in temperature (Fig. 8a, b), the cooling due to the ACI of sulfate causes a marked increase in the pressure over the North and Center ECMR (Fig. 8c). However, the pressure of the adjacent ocean does not significantly change, which may result in a decrease in the land-sea pressure gradient. The variation in the land-sea pressure gradient caused by the increase in sea level pressure further leads to changes in the surface meridional wind and circulation, which, in turn, affects the intensity of the EASM and precipitation.

### The changes of East Asian summer monsoon and precipitation

Figure 9a shows the changes in the 850 hPa wind field and meridional wind caused by the ACI of sulfate. The ACI of sulfate causes a significant weakening of the southerly wind over the ECMR during summer, especially in the Center and North ECMR, as demonstrated in Fig. 9a and Table 2. Combined with the changes in the meridional wind and vertical velocity (Fig. 6), we observe that the ACI of sulfate leads to a marked weakening of the low-level southerly wind and high-level northerly wind. This also results in an intensification of the ascending motion over the tropical ocean (south of 20° N) and a weakened ascending motion over the South and North ECMR. Thus, the ACI of sulfate leads to a weakening of the EASM.

Figure 9b shows the changes in the total precipitation rate and convective precipitation rate caused by the ACI of sulfate. Based on the weakening of the EASM, the ACI of sulfate results in a significant decrease in the total precipitation rate in the South ECMR, the precipitation rate in the Center ECMR exhibits an insignificant increase, the North ECMR shows a slight decrease. This implies that the weakened EASM reduces water vapor and convective movements in the South ECMR. Further analysis of the contribution between the convective precipitation rate and large-scale precipitation rate suggests that, as shown in Fig. 9b, the significant change in the total precipitation rate in the South ECMR is mainly due to the change in the convective precipitation rate (71%), which is mainly caused by variations in convective activities (Fig. 6c) induced by a weakened EASM. The contribution of the large-scale precipitation rate is 29% and relatively small.

## Conclusions and discussion

In this study, the ACI of sulfates on summertime clouds, the EASM, and precipitation over the ECMR were investigated with the NCAR Community Atmospheric Model version 5 (CAM5) by halving the sulfate aerosol concentration involved in the nucleation process. The model captures the main circulation and precipitation features of the EASM. In summer, the distribution of the CCN is consistent with the concentration of sulfate aerosol, mainly distributed over the ECMR. The high value is mainly distributed below 700 hPa while the maximum values occurs below ~ 900 hPa to the north of 30°N. The ACI of sulfate caused a significant impact on the CCN (increased by ~ 30%), mainly in the Center and North ECMR. However, the relationship between sulfate aerosol and CDNC also needs consider the conditions of water vapor and supersaturation^40^. The increase in the in-cloud CDNC (~ 34%) caused by the ACI of sulfate results in a decrease in the Reff (~ 8%) below 850 hPa over the South ECMR, leading to an increase in the LWP by 11% over the ECMR. The change in the SWCF is − 3.18 W·m^−2^. The ACI of sulfate causes the surface air temperature to drop by 0.32 K and the atmospheric temperature in the middle and upper troposphere to reduce by ~ 0.3 K. Increased cooling occurs in the North and Center ECMR. This observation is consistent with results reported in Jiang^22,24^. The change in temperature mainly depends on the net shortwave and latent heat fluxes. However, the diabatic heating term cannot completely explain this change, which may also be affected by temperature advection caused by changes in circulation. Cooling caused by the ACI of sulfate causes a positive anomaly in the sea level pressure and, thus, a weakening of the EASM averaged over the ECMR, as well as a decrease in the precipitation over the South ECMR. Convective (large-scale) precipitation rate changes contribute 71% (29%) to total precipitation rate changes. In summary, the ACI of sulfate aerosol will cause changes in cloud characteristics, leading to an enhancement of SWCF (more negative) and atmosphere temperature cooling. This results in positive anomalies in sea level pressure, which causes the EASM to weaken in the ECMR and reductions in the precipitation in the South ECMR. The ACI of sulfate may be one of the contributors to the weakening of the EASM in the late 1970s.

The uncertainties in quantifying the ACI through GCMs stem largely from two sources: (1) our limited understanding of aerosol and cloud processes and (2) the change in the aerosol state itself from pre-industrial to present-day is uncertain, which is caused by uncertainties in aerosol emissions and atmospheric aerosol processes^41^. Therefore, we used an experimental design in this study that distinguishes the mechanism of the ACI of sulfate on EASM, while retaining the ARI. Under this experimental design, the change in the radiation forcing caused by the ACI is similar to the ARI of sulfate, whose influences on the EASM are comparable. In addition, we also designed a set of tests for cutting the concentration of sulfate aerosols by 100% in the nucleation calculation. We found that the increased CCN is nearly equal to the entirety over the ECMR in summer. Sulfate aerosols account for approximately 85% of the contribution, indicating an absolute dominance in the formation of CCN in East Asia^5^. The sign and distribution of the changes in the resulting CDNC, Re, LWP, and SWCF are identical to the results between the CTRL and SENS, but the value is approximately 3 times larger. More SWCF changes lead to more changes in surface air temperature (− 0.85 K) and sea level pressure (0.79 hPa). This led to a weakened monsoon intensity (− 0.31 m·s^−1^), and increased reduction of precipitation (− 0.54 mm·d^−1^), mainly in the South ECMR. However, the experimental design used in this study does not fully represent realistic scenarios and requires further improvement. Although our results show that the ACI of sulfate aerosols leads to a weakening of monsoon and a decrease of precipitation (71% in convective precipitation), it is mainly through the feedback of circulation. The use of CAM5 to simulate ACI bears certain limitations. For example, the effect that aerosol has on convective clouds is not included^22^, the total dispersion effects on both the Re and Au are not fully considered^42^, and ice nuclei concentrations are not a function of temperature and not coupled to aerosol characteristics^41^. Previous studies^24,42^ have shown that the ACI in the cloud-resolving models can enhance convective precipitation by invigorating convections. Further studies with improved models are needed, but are beyond the scope of this study. Although a 2-year low-pass filter was used in this study to remove inter-annual changes, there is still a large amount of uncertainty due to large cloud uncertainty, which requires a GCM to more accurately simulate clouds. In addition, experiments that only consider the rapid response of ACI also have certain limitations. The responses of SST to aerosol changes is also important, which affect the land-sea thermal contrast and subsequent monsoon circulation^43–45^. However, studies have also shown that the fast response makes a greater contribution to changes in monsoon intensity and precipitation over land north of 20°N, while the slow response has a greater impact on ocean changes^45^. The focal point of this study is the influence mechanism of the ACI on the monsoon circulation and precipitation in the ECMR, which is also the basis for distinguishing the contribution of the ECMR to the fast and slow responses of ACI. Aerosol effects with coupled simulations will be investigated in future studies to understand the role of air-sea coupling.

## Methods

### Model description

The CAM5 used in this study was the atmospheric component of the Community Earth System Model (CESM), which was developed by National Center for Atmospheric Research (NCAR)^46^. The CAM5 model included the chemistry and aerosol modules (Model for OZone And Related chemical Tracers, MOZART)^47^, radiation transport mechanism (Rapid Radiative Transfer Method for GCMs)^48^, and cloud macroscopic^49^ and microphysical mechanisms^50,51^. More details about this model can be found in Jiang^22^ and Wang^25^. The ARI, semi-direct effect, and ACI for liquid phase clouds were included in the model^27^, as well as the interaction between aerosol and atmosphere^24^. The sources of aerosol emissions were obtained from the emissions inventory reported in Emmons^47^.

As previously mentioned, a two-moment bulk cloud microphysical scheme was employed in the model to describe the mass and number concentrations of cloud droplets. According to Abdul-Razzak and Ghan^40^, the following formula can be used to calculate the activated aerosol number concentration (*N*):

1 $$ N = \sum\limits_{i = 1}^{I} {N_{i} \frac{1}{2}} \left[ {1 - erf\left( {u_{i} } \right)} \right], $$

where *u* is related to the activated aerosol’s radius and supersaturation, which is consistent with the description of the Köhler equilibrium equation. *N*_*i*_ is the aerosol number concentration of each hygroscopic aerosol species.

The cloud droplet effective radius, r_e_, is given by the following equation:

2 $$ r_{e} = \frac{{\Gamma \left( {\mu + 4} \right)}}{{2\lambda \Gamma \left( {\mu + 3} \right)}}, $$

where $\Gamma$ is the Euler gamma function, *λ* is the slope parameter, and *µ* = 1/*η*^2^ − 1 is the spectra shape parameter. Here, *η* is the relative radius dispersion of the size distribution, which can be specified following Martin^52^:

3 $$ \eta = 0.0005714N_{c}^{\prime \prime } + 0.2714, $$

where *N*_*c*_*′′* is the number concentration of in-cloud droplets in cm^−3^, which is the product of the total liquid cloud number concentration and the liquid cloud fraction.

### Numerical experiment design

The CAM5 model uses the MOZART chemistry mechanism with a horizontal resolution at a latitude and longitude of 1.9° and 2.5°, respectively, with 30 vertical levels. The boundary configurations of CAM5 are kept at the level of the preset-day (year 2000), including SST, GHGs, and aerosol emissions. Each experiment was run for 31 model years representing the present-day climate, but only the last 30 years of the simulations were used in this study.

Our aim was to explore the ACI of sulfate on the EASM. To obtain the ACI of sulfate, two numerical experiments were conducted. In the control experiment (CTRL), all types of the ARI and ACI radiative processes were included. According to the total SO_2_ emissions by decade and region given by Smith^1^, we calculated that the emissions in East Asia (China and Japan) from 1950 to 1970s were about 25,282 Gg·SO_2_, and the emissions from 1980 to 2000s were about 53,745 Gg·SO_2_, which increased by about 100%. Therefore, according to Eq. (1), the number concentration of sulfate in the nucleation process was reduced by 50% in the sensitivity test (SENS), which represented the situation from 1950 to 1970s. In this process, all other parameters were identical, and the emission source remained unchanged, with a theoretically unchanged the ARI. The differences between the results of the CTRL and SENS were regarded as the ACI of sulfate on the climate over the ECMR. It is expected that the design of the sensitivity experiment will be used to explore whether the ACI of sulfate is one of the possible reasons for EASM weakening.

As the first step to understand the ACI of sulfate and its impacts on the eastern China climate, CAM5 was run with the prescribed sea surface temperature (SST) and sea ice based on the experiments conducted under the Atmospheric Model Intercomparison Project (AMIP)^53^, where the (slow) SST response was not taken into account. While our sensitivity experiment highlights the ACI of sulfate at extreme levels, this does not represent the actual situation. A *t* test was used to assess the significance of these differences. Similar to Song^17^, a 2-year low-pass filter was applied to suppress interannual variability.

## Data Availability

The datasets generated and/or analyzed during the current study can be obtained from the corresponding authors.
